# Development and Evaluation of a High Density Genotyping ‘Axiom_*Arachis*’ Array with 58 K SNPs for Accelerating Genetics and Breeding in Groundnut

**DOI:** 10.1038/srep40577

**Published:** 2017-01-16

**Authors:** Manish K. Pandey, Gaurav Agarwal, Sandip M. Kale, Josh Clevenger, Spurthi N. Nayak, Manda Sriswathi, Annapurna Chitikineni, Carolina Chavarro, Xiaoping Chen, Hari D. Upadhyaya, Manish K. Vishwakarma, Soraya Leal-Bertioli, Xuanqiang Liang, David J. Bertioli, Baozhu Guo, Scott A. Jackson, Peggy Ozias-Akins, Rajeev K. Varshney

**Affiliations:** 1 International Crops Research Institute for the Semi-Arid Tropics (ICRISAT), Hyderabad, India; 2 University of Georgia (UGA), Tifton, USA; 3 Center for Applied Genetic Technologies, University of Georgia (UGA), Athens, USA; 4 Crops Research Institute (CRI), Guangdong Academy of Agricultural Sciences (GAAS), Guangzhou, China; 5 Crop Protection and Management Research Unit, USDA-ARS, Tifton, USA; 6 The University of Western Australia, Crawley, Australia

## Abstract

Single nucleotide polymorphisms (SNPs) are the most abundant DNA sequence variation in the genomes which can be used to associate genotypic variation to the phenotype. Therefore, availability of a high-density SNP array with uniform genome coverage can advance genetic studies and breeding applications. Here we report the development of a high-density SNP array ‘Axiom*_Arachis*’ with 58 K SNPs and its utility in groundnut genetic diversity study. In this context, from a total of 163,782 SNPs derived from DNA resequencing and RNA-sequencing of 41 groundnut accessions and wild diploid ancestors, a total of 58,233 unique and informative SNPs were selected for developing the array. In addition to cultivated groundnuts (*Arachis hypogaea*), fair representation was kept for other diploids (*A. duranensis, A. stenosperma, A. cardenasii, A. magna* and *A. batizocoi*). Genotyping of the groundnut ‘Reference Set’ containing 300 genotypes identified 44,424 polymorphic SNPs and genetic diversity analysis provided in-depth insights into the genetic architecture of this material. The availability of the high-density SNP array ‘Axiom*_Arachis*’ with 58 K SNPs will accelerate the process of high resolution trait genetics and molecular breeding in cultivated groundnut.

Crop improvement programs in general are focused on enhancing productivity, improving quality and resilience to biotic and abiotic stress by creating and/or harnessing genetic diversity. Genomics-assisted breeding (GAB) has accelerated crop improvement programs for development of improved cultivars in several crops^1^. Availability of high density genotyping platform with uniformly distributed genome-wide genetic markers is must have genomic resource in a crop for high resolution genetic dissection of complex traits and tracking the favorable alleles in a breeding population^2^.

Single nucleotide polymorphisms (SNPs) are the most abundant DNA sequence variations among various types of structural/genetic/sequence variations in the genome. Until recently, it has been a tedious, labor-intensive and expensive task to develop even a limited number of SNPs. In the last decade, next-generation sequencing (NGS) technologies have evolved very rapidly and have become the cheapest and fastest method of identification of genome-wide SNPs^1^. The most commonly used NGS approach for identifying and assaying SNPs is genotyping-by-sequencing (GBS)^3^. While GBS provides generation of high-density SNP data in less time and less cost, allelic data are not generated for all the SNPs detected among individuals/lines in a given population^4^. Furthermore, though the imputation methods are available to infer missing data, these methods rely on prior extensive genotyping data.

High-density fixed SNP arrays, though expensive as compared to GBS, provide genotyping data for almost all SNPs for all the individuals in a population. There are several genetics and breeding methods e.g. genomic selection that require consistent genotyping data for the same SNP loci across different germplasm sets and over filial generations in breeding. Therefore, fixed SNP arrays with >40 K SNPs have been developed and used for a variety of genetics and breeding applications in several crops such as rice^5^,^6^, maize^7^,^8^, sunflower^9^, soybean^10^, oat^11^, cotton^12^, and wheat^13^,^14^.

Groundnut or peanut (*Arachis hypogaea*) is a globally important crop that is cultivated in >100 countries and consumed in almost every country. The last decade has witnessed a steady increase in demand due to a tremendous population growth in Asia and Africa. In 2014, this crop produced 42.3 Mt from 25.7 Mha with the average productivity of 1649 Kg/ha (http://faostat.fao.org/) but there is an existential need to increase its productivity to meet the growing demand. Cultivated groundnut is an allotetraploid (2*n* = 4*x* = 40) species with two subgenomes (A and B) that complicates sharing of diversity between cultivated species and wild diploid species^15^. To facilitate genetics, breeding as well as evolutionary biology studies, genomic resources such as molecular markers, genetic maps, cytogenetic maps, etc. have been developed in both diploid as well as tetraploid species^2^,^16^. Availability of draft genome sequences for both ancestral species of cultivated groundnut namely *A. duranensis* (A subgenome) and *A. ipaensis* (B subgenome) in 2016^17^,^18^ is a major boost for the global groundnut research community.

With the availability of draft genome sequences, re-sequencing and transcriptome sequencing of several accessions of tetraploid species as well as a number of diploid species accessions, we identified a large number of SNPs and selected a comprehensive set of informative genome-wide SNPs. We report here selection and development of array with 58 K SNPs as well as its validation and utility in genetic diversity analysis in groundnut.

## Results

### SNP selection and array design

**Figure 1:**
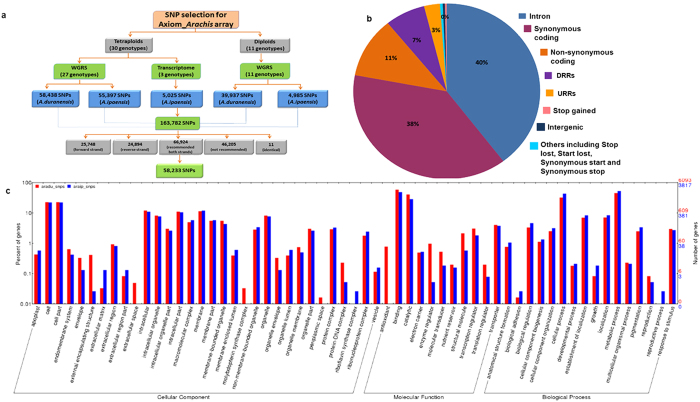
Selection of 58,233 SNPs from *Arachis* genome for developing Axiom_*Arachis* SNP array and their genomic features. This illustration shows (**a**) the type of genotypes and sequencing data used for identifying large scale genome-wide SNPs and the final selection of high quality SNPs used in designing SNP array, (**b**) genomic positions of selected SNPs and (**c**) annotation of selected 58 K SNPs.

The analysis of the sequencing data generated from 41 genotypes (30 tetraploids and 11 diploids) against the genomes of two groundnut progenitors i.e., *A. duranensis* (A subgenome) and *A. ipaensis* (B subgenome) (Supplementary Table S1) yielded a total of 163,782 SNPs i.e., 98,375 SNPs from A subgenome and 65,407 SNPs from B subgenome (Fig. 1a). Of the 41 genotypes, sequence analysis of 30 tetraploid genotypes identified 118,860 SNPs (58,438 SNPs from A subgenome and 60,422 SNPs from B subgenome) while 11 diploid genotypes yielded 44,922 SNPs (39,937 SNPs from A subgenome and 4,985 SNPs from B subgenome). Among 30 tetraploid genotypes, analysis of WGRS data for 27 genotypes yielded 113, 835 SNPs (58,438 SNPs from A subgenome and 55,397 SNPs from B subgenome) and RNAseq data of three tetraploid genotypes yielded 5,025 SNPs from B subgenome.

All the identified 163,782 SNPs were subjected to filtering to select SNPs of good quality. The above SNP set also had 52 highly informative SNPs associated with resistance to foliar fungal diseases and oil quality. During filtering process, a total of 96,858 SNPs were discarded as 46,205 SNPs were found on both genomes, 50,642 SNPs were present on either of the two strands of DNA, and 11 SNPs were found identical. As a result, only 66,924 SNPs passed the filtering test (Fig. 1a). From this set, 825 SNPs, however, were further removed because of ambiguity and multi-allelic nature of these SNPs, leaving 66,099 good quality SNPs. From the set of 66,099 good quality SNPs, finally, 58,233 SNPs uniformly distributed across A and B subgenomes were tiled on the Axiom_*Arachis* array (Supplementary Table S2).

### Genomic position and functional annotation of selected SNPs

With respect to genomic positions of the SNPs fixed on Axiom_*Arachis* array, a total of 22,224 and 23,222 SNPs have come from synonymous coding and intronic regions, respectively (Table 1, Fig. 1b). The other major groups include non-synonymous coding (6,486), UTR_3_prime (4,027) and UTR_5_prime (1,597). In the case of A subgenome, maximum SNPs were located in synonymous_coding (12,087) followed by intronic (11,236), non_synonymous_coding (3,462), UTR_3_prime (2,038) and UTR_5_prime (923) (Table 1). Similarly in the case of B subgenome, maximum SNPs were intronic (11,986) followed by synonymous_coding (10,137), non_synonymous_coding (3,024), UTR_3_prime (1,989) and UTR_5_prime (674) (Table 1).

The functional annotation information was used to categorize the SNPs into different categories i.e., biological processes, molecular function, and cellular component (Fig. 1c). A majority of the SNPs found in genes were classified into cellular component followed by biological process and molecular function. SNPs underlying the genes coding for extracellular, periplasmic space proteins and involved in antioxidant activity were specifically found to be enriched in *A. duranensis* genome but not in *A. ipaensis* genome. On the other hand, genes involved in reproductive processes and riboflavin synthase complex were enriched in the *A. ipaensis* genome but not in the *A. duranensis* genome. Cell and cell part, binding and catalytic activity, and cellular and metabolic process were the most representative terms in cellular component, molecular function and biological process category.

### Genome-wide distribution of selected SNPs

Selected 58,233 SNPs had good representation from both subgenomes of tetraploid groundnut. Of the 58,233 SNPs, 29,983 SNPs have come from A subgenome while 28,250 SNPs from B subgenome and achieved coverage of 2,912 SNPs per pseudomolecule (Table 2). An average 2,998 SNPs per pseudomolecule were selected from A subgenome and the number of SNPs ranged from 2,303 (pseudomolecule A07) to 4,714 (pseudomolecule A01). Similarly B subgenome had an average of 2,825 SNPs per pseudomolecule and ranged from 2,405 (pseudomolecule B01) to 3,443 (pseudomolecule B03). In terms of source for 58,233 SNPs, 44,501 SNPs were selected by comparing both the genome assemblies with tetraploid genotypes, 21 trait linked SNPs for foliar disease resistance and oil quality and 13,732 SNPs with diploid genotypes. Of the 13,732 SNPs, 2,195 SNPs from *A. cardenasii*, 3,834 SNPs from *A. duranensis*, 2,389 SNPs from *A. stenosperma*, 2,605 SNPs from *A. magna* and 2,709 SNPs from *A. batizocoi* were identified (Table 3, Supplementary Table S3).

### Polymorphism analysis in the ‘Reference Set’

**Figure 2:**
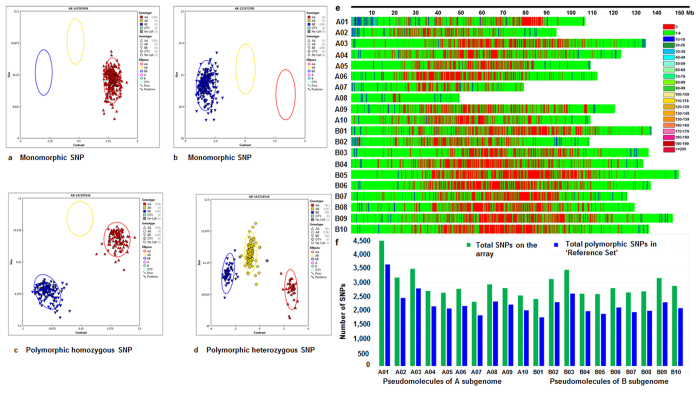
SNP calling pattern, genome density and polymorphism of SNPs in ‘Reference Set’ using Axiom_*Arachis* SNP array. This figure shows (**a**,**b**) monomorphic SNPs identified in the genotyping data, (**c**) polymorphic SNPs without heterozygosity i.e., homozygous SNPs, (**d**) polymorphic SNPs with heterozygosity, (**e**) genome-wide distribution of SNPs, and (**f**) pseudomolecule-wise distribution of SNPs on array and polymorphic SNPs in the ‘Reference Set’.

Axiom*_Arachis* SNP array was used to genotype the ‘Reference Set’ comprising of 300 genotypes developed by International Crops Research Institute for the Semi-Arid Tropics (ICRISAT) (Fig. 2a–e, Supplementary Table S4). Genotyping of this set with the SNP array resulted in generation of genotyping data for 58,233 SNPs on 297 genotypes as QC for allele call failed for three samples. Upon identifying the polymorphic SNPs separately for the diploid species genotypes and tetraploid species genotypes, 40,714 polymorphic SNPs were identified on a panel of 36 wild genotypes while 9,312 polymorphic SNPs in the set of 264 cultivated tetraploid genotypes. Comparison of SNPs identified in the above two sets resulted in the identification of 5,625 common SNPs (Table 2). Subsequently, 44,424 polymorphic SNPs were identified through combined analysis of all the 297 genotypes of the ‘Reference Set’ and were used for further genetic analysis.

**Figure 3:**
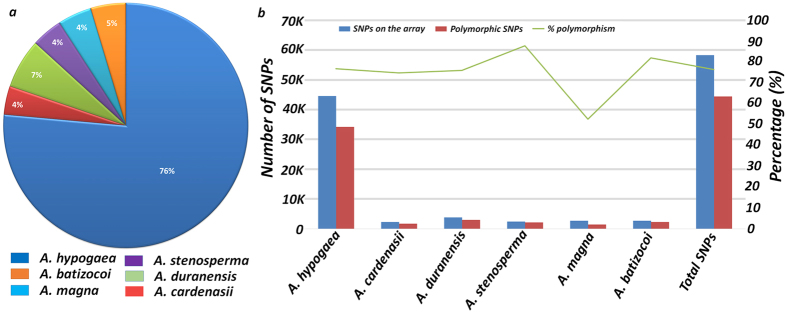
Species representation of SNPs on the Axiom_*Arachis* array and their polymorphism status in the ‘Reference Set’. This figure shows (**a**) percentage (%) of SNPs selected from different species using sequencing data for development of array, and (**b**) percentage (%) of polymorphism achieved by these SNPs in the ‘Reference Set’.

Of the 44,424 polymorphic SNPs, 23,559 SNPs were from A subgenome while 20,865 SNPs from B subgenome and achieved an average of 2,221 polymorphic SNPs per pseudomolecule (Fig. 2f, Table 2). An average 2,356 polymorphic SNPs per pseudomolecule were from A subgenome and the number of SNPs ranged from 1,822 SNPs (pseudomolecule A07) to 3,631 SNPs (pseudomolecule A01). Similarly B subgenome had an average 2,087 polymorphic SNPs per pseudomolecule and it ranged from 1,750 (pseudomolecule B01) to 2,598 (pseudomolecule B03). Further, SNPs selected from *A. stenosperma* (87.7%) and *A. batizocoi* (82.1%) showed highest level of polymorphism while SNPs from *A. magna* (52.5%) showed minimum polymorphism in the ‘Reference Set’ (Table 3). The SNPs from *A. hypogaea* (76.8%), *A. cardenasii* (74.9%) and *A. duranensis* (76.0%) showed similar level of polymorphism (Fig. 3).

Major allele frequency for the polymorphic SNPs in the ‘Reference Set’ ranged from 0.50 (52 SNPs) to 0.99 (6,853 SNPs) with an average of 0.92 (Supplementary Table S5). Minor allele frequency ranged from zero (1,814 SNPs) to 0.50 (52 SNPs) with an estimated average of 0.08. Similarly, heterozygosity in the population ranged from zero (7,842 SNPs) to 0.87 (AX-147231295) with estimated average of 0.02. Polymorphic information content (PIC) value for SNPs on the array ranged from 0.01 (5,420 SNPs) to 0.50 (608 SNPs) with an estimated average of 0.13 (Supplementary Table S5) in the population.

### Genetic analysis of the ‘Reference Set’

**Figure 4:**
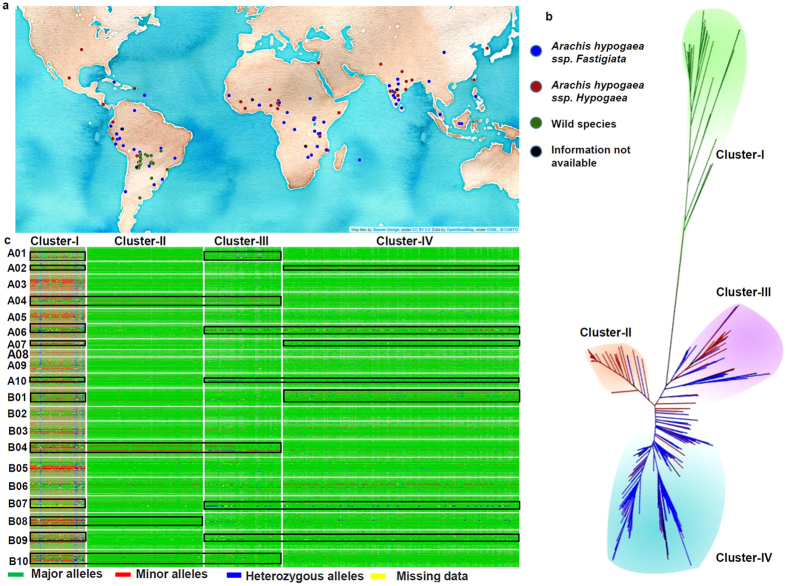
Genetic diversity in ‘Reference Set’ using Axiom_*Arachis* SNP array. This figure shows (**a**) global distribution of genotypes of the ‘Reference Set’. The world map was constructed using cartoDB (https://fee.carto.com/) with OpenStreetMap data (https://www.openstreetmap.org/). (**b**) Grouping pattern of the ‘Reference Set’ genotypes based on polymorphic SNPs, and (**c**) genome architecture pattern among different clusters.

Generation of high throughput SNP genotyping data on the ‘Reference Set’ provided an opportunity to gain deeper insights into the genetic relatedness among the genotypes and also the genetic architecture of this important germplasm set (Fig. 4a). The genetic diversity analysis with 44,424 polymorphic SNPs identified four clusters (Cluster-I, Cluster-II, Cluster-III and Cluster-IV) (Fig. 4b, Supplementary Table S6). The Cluster-I consisted of genotypes of the diploid (wild) species while Cluster-II, Cluster-III and Cluster-IV consisted of tetraploid genotypes (*A. hypogaea*). Among the tetraploid groups, the Cluster-II had genotypes form *hypogaea* subspecies, Cluster-III had both the subspecies i.e., *hypogaea* and *fastigiata* while Cluster-IV had genotypes from *fastigiata* subspecies. The diverse lines with maximum genetic distance within cultivated groups can be used for developing genetic and breeding populations for both mapping traits as well as for developing improved varieties with desirable agronomic traits and enhanced genetic base.

This array has also shown significant loss of diversity in the cultivated gene pool and preferential selection of genomic regions in these subspecies (Fig. 4c). The loss of genetic diversity is clearly visible in cultivated genotypes i.e., three clusters (Cluster-II, III and IV) upon comparison with wild species accessions grouped together in Cluster-I. Most importantly, few genomic regions were found conserved and were specific to subspecies *fastigiata* and *hypogaea* of the *A. hypogaea* (cultivated tetraploid). For example, the genomic regions conserved to subspecies *fastigiata* were observed on pseudomolecules A02, A06, A07 and A10 of A subgenome while B01, B07 and B09 of B subgenome. Similarly, the genomic regions conserved to subspecies *hypogaea* were observed in pseudomolecules A04 of A subgenome while B04, B08 and B10 of B subgenome.

### Identification of subspecies specific high frequency SNPs

**Figure 5:**
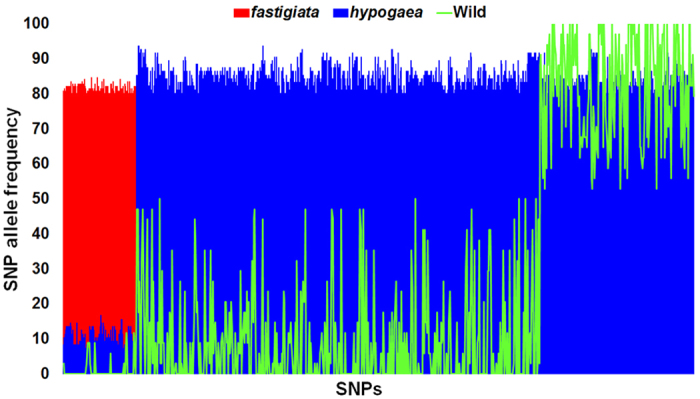
Allele frequencies for subspecies *hypogaea* specific SNPs in two subspecies of cultivated groundnut and wild accessions. The frequency of alleles at each SNP locus were calculated and combined chart was prepared using alternate allele frequencies of SNPs with contrasting allele frequencies in three categories, *fastigiata, hypogaea* and wild. Each bar represents the alternate allele frequency observed at particular SNP site in three categories. The red, blue and green bar/line indicates the alternate allele frequencies observed in *fastigiata, hypogaea* and wild categories, respectively.

We have identified the pseudomolecule-wise distribution of subspecies specific high frequency (>80%) SNPs for subspecies *fastigiata* and *hypogaea* (Fig. 5, Supplementary Table S7). Of the total identified 809 high frequency SNPs, 94 SNPs were from subspecies *fastigiata* and 517 SNPs from subspecies *hypogaea*. No common SNP was detected between subspecies *fastigiata* and wild accessions, while, 198 SNPs were found common between subspecies *hypogaea* and wild accessions. The prediction of effect for these 198 SNPs indicated their location in 133 genes having missense or nonsense mutations. The functions of these genes and their association in various biological functions has been described in the Supplementary Table S8. The enrichment analysis using GO ids showed that majority of genes have binding, catalytic and transporter functions and are involved in catalytic and cellular processes (Supplementary Figure S1).

## Discussion

### High density genotyping ‘Axiom_*Arachis*’ array

A high density SNP genotyping array with uniform genome coverage is must in any crop for conducting high resolution trait mapping^2^,^19^. Development of SNP array for high throughput genotyping was very much required in the case of groundnut due to its large genome size and low genetic diversity in the cultivated gene pool^2^,^16^,^19^. Availability of Axiom_*Arachis* array with 58,233 SNPs to the *Arachis* community provides an opportunity to generate high throughput genotyping data on different types of genetic and breeding populations for accelerating genetic diversity, high resolution trait mapping and breeding applications. Similar arrays were developed recently in other crop species such as rice (44 K by McCouch *et al*.^5^; 50 K by Chen *et al*.^6^; 50 K by Singh *et al*.^20^), sunflower (11 K SNPs by Bachlava *et al*.^9^), soybean (50 K SNPs by Song *et al*.^10^), oil palm (171 K SNPs by Kwong *et al*.^21^), maize (58 K SNPs by Ganal *et al*.^7^) and wheat (90 K SNPs by Wang *et al*.^13^). Much higher density genotyping arrays are available in animal species like chicken (600 K SNPs by Kranis *et al*.^22^), cattle (648 K by Rincon *et al*.^23^) and human (900 K SNPs by Kathiresan *et al*.^24^). In the case of plants, 819 K SNPs arrays for wheat^14^ and 600 K SNPs arrays for maize^8^ are the most-dense publicly available genotyping arrays.

### Fair representation of genome and *Arachis* species

The genome size of A and B subgenomes is reported to be 1,070 Mb and 1,360 Mb, respectively^17^. Axiom_*Arachis* array developed in this study has fair genome representation *i.e*., 51.5% from A subgenome and 48.5% from B subgenome with an average 2,998 and 2,825 SNPs per pseudomolecule for A and B subgenome, respectively. Also this array achieved high density genome coverage of 1 SNP per 42 Kb in tetraploid genome while 1 SNP per 36 Kb in A subgenome and 1 SNP per 48 Kb in B subgenome. The above density is comparable to other recently developed SNP arrays in maize^7^, rice^6^ and oil palm^21^.

We also tried to make this array diverse by including informative SNPs from different sources i.e., 76.7% SNPs from *A. hypogaea* (cultivated tetraploid genotypes), 6.2% SNPs from *A. duranensis*, 4.7% SNPs from *A. batizocoi*, 4.5% SNPs from *A. magna*, 4.1% from *A. stenosperma*, and 3.8% SNPs from *A. cardenasii*. The 58,233 SNPs placed on the newly developed array represented mostly intronic region with 39.8% share followed by 38.1% synonymous_coding, 11.1% non-synonymous_coding, 6.9% UTR_3_prime and 2.7% UTR_5_prime. The remaining 1.2% SNPs included intergenic, stop_gained, stop_lost, non-synonymous_start, non-synonymous_stop, start_lost, and others. The 50 K SNP array developed by Singh *et al*.^20^ in rice also had large proportion of SNPs from intronic region (41% SNPs) followed by non-synonymous_coding (20%), synonymous_coding (18% SNPs), UTR_3_prime (14% SNPs) and UTR_5_prime (7% SNPs). Functional annotation of SNPs from two subgenomes indicated *A. duranensis* enriched with genes coding for extracellular, periplasmic space proteins and involved in antioxidant activity while *A. ipaensis* with genes involved in reproductive processes and riboflavin synthase complex.

### Insights on genetic diversity and genetic relationship

The newly developed Axiom*_Arachis* SNP array was deployed to study genetic diversity and genetic relatedness in the ‘Reference Set’ developed by ICRISAT^25^. This set has 300 individuals of which 264 are cultivated tetraploid while 36 are wild accessions. Out of 36 wild accessions, 34 were diploid while two accessions were tetraploid belonging to *A. monticola*. High quality SNP genotyping data was generated successfully for all except three genotypes of the panel mainly due to poor QC for allele call. The polymorphism rate was four times higher in the smaller set of wild genotypes than the larger set of cultivated genotypes with mere ~10% common SNPs between both the sets.

After removing the common SNPs, 77.6% SNPs showed polymorphism in the ‘Reference Set’ with comparatively higher rate of polymorphism in A subgenome (53.0%) than the B subgenome (47%). Considering the genome size of A (1,070 Mb) and B (1,360 Mb) subgenomes, single SNP per 45 Kb polymorphism density has been achieved in A subgenome as compared to single SNP per 65 Kb in B subgenome. Further, SNPs selected from *A. stenosperma* (87.7%) and *A. batizocoi* (82.1%) showed highest polymorphism as compared to *A. hypogaea* (76.8%), *A. duranensis* (76.0%), *A. cardenasii* (74.9%) and *A. magna* (52.5%).

Mean major allele frequency, minor allele frequency, heterozygosity and PIC was found to be 0.92, 0.08, 0.02 and 0.13, respectively in the ‘Reference Set’. Phylogenetic analysis clearly grouped all the genotypes of the ‘Reference Set’ in four groups. As expected, the wild genotypes were grouped together in Cluster-I while cultivated genotypes (*A. hypogaea*) were clustered into three distinct groups. Majority of the genotypes from *hypogaea* subspecies were grouped together in Cluster-II, both the subspecies i.e., *hypogaea* and *fastigiata* genotypes in Cluster-III and genotypes of *fastigiata* subspecies in Cluster-IV. The grouping of different subspecies genotypes using the SNP array was found much better than the earlier studies conducted with SSR and DArT genotyping on the same germplasm set^26^,^27^.

### Conserved genomic regions harboring domestication related genes

The cultivated groundnut crop across the world can be divided into four market types from two subspecies. The subspecies *A. hypogaea hypogaea* do not flower on main stem, have alternate branching patterns, mature later and produce large seeds while *A. hypogaea fastigiata* produce flowers on the main stem, have sequential branching patterns, mature earlier and produce smaller seeds^28^. This array has demonstrated immense power not only in grouping the genotypes of different subspecies of *A. hypogaea* but also showing preferential selection of genomic regions in these subspecies. Significant loss of diversity can be clearly observed in all three clusters representing cultivated tetraploid genotypes as compared to the cluster representing wild species accessions. This study indicated that during the evolution of subspecies *fastigiata* and *hypogaea* of the *A. hypogaea* (cultivated tetraploid), selective genomic regions remained conserved. These genomic regions might be harboring genes that are responsible for maintaining subspecies specific features.

In order to get further insights, we identified the pseudomolecule-wise subspecies specific high frequency SNPs for *fastigiata* and *hypogaea*. Prediction of 198 common SNPs between subspecies *hypogaea* and wild accessions indicated their location in 133 genes including plant defense against biotic and abiotic stresses, cellular growth and development, seed and pollen development. More importantly genes related to domestication traits such as skotomorphogenesis, flowering time (*Aradu.1A8NN.1*), seed maturity and germination (*Aradu.PZ509.1*), lateral root development (*Aradu.895HT.1*), stem elongation (*Aradu.Y6SZD.1*) and self-incompatibility (*Araip.D2CP3.1*) have shown genomic variation between these two subspecies. Such variation for domestication related traits might be playing an important role in retaining the basic features of these two subspecies during the course of evolution.

In summary, the present study reports development of Axiom_*Arachis* array with 58 K informative SNPs and its successful deployment in understanding the genetic diversity of ICRISAT ‘Reference Set’ in groundnut. This array is an important genomic resource for the *Arachis* and especially the groundnut community that will be useful not only for accelerating genetics and breeding applications but also to understand evolutionary biology in *Arachis* species.

## Materials and Methods

### Plant materials

A total of 41 *Arachis* spp. accessions (25 from University of Georgia, USA; 15 from ICRISAT, India and one from Crops Research Institute-Guangdong Academy of Agricultural Sciences, Guangzhou, China) were used for generating the sequence data in earlier studies^18^,^29^ and the same data was used for identification of SNPs. Of the 41 accessions, 30 genotypes represented cultivated tetraploid species and 11 wild accessions represented 6 different diploid species.

A total of 38 accessions were used for whole genome re-sequencing (WGRS) while 3 accessions (ICGV 91114, JL 24 and J 11, all tetraploids) were used for transcriptome sequencing. 38 accessions used for WGRS included 23 tetraploids, 4 tetraploid pooled samples (resistant and susceptible for foliar disease resistance) and 11 diploid species genotypes representing *A. duranensis* (PI 475845, ICG 8138 and ICG 8123), *A. ipaensis* (ICG 8206), *A. batizocoi* (ICG 8209, ICG 13160 and K9484), *A. magna* (ICG 8960 and KG30097), *A. stenosperma* (V10309) and *A. cardenasii*. The ‘Reference Set’ that is comprised of 300 accessions coming from 48 countries^25^ including 36 wild species accessions was used for genotyping with SNP array.

### DNA/RNA isolation, sequencing and SNP identification

High quality DNA isolation using modified CTAB-based method followed by quantification and quality check of DNA was done as mentioned in Mace *et al*.^30^. The WGRS data were generated for 21 tetraploid accessions and 4 diploids at UGA, USA; and 6 tetraploids (TAG 24, GPBD 4 and 4 tetraploid pooled samples for foliar disease resistance) at ICRISAT, India. The RNA-seq data for 3 tetraploid genotypes (ICGV 91114, JL 24 and J 11) were also generated at ICRISAT, India. The WGRS data for 7 diploids (PI 475845, ICG 8138, ICG 8960, ICG 8209, ICG 13160, ICG 8206 and ICG 8123) were generated at Macrogen Inc., South Korea.

The raw sequences obtained were filtered using various softwares to get high quality reads for downstream processing. Briefly, the adapter sequences were trimmed using Cutadapt v1.2.1^31^ while quality trimming was carried out using TrimGalore v0.3.7 software. Such high quality sequences were mapped against the two diploid genomes (A and B subgenomes represented by *A. duranensis* and *A. ipaensis*)^17^ with Bowtie2^32^ and SNPs were identified. Further, the homeologous SNPs were removed using SWEEP Prime version program^33^. Subsequently, SNPs which were present within 10 kb of *A. duranensis* and *A. ipaensis* annotated genes^17^ were used for downstream processing. The 35 bp sequences flanking to both side of selected SNPs were extracted using custom script and searched against A and B subgenomes for uniqueness using BLASTN program. Finally, the SNPs showing unique hit of ≥94% identity or across at least 60 aligned bases were selected for array development.

### Array design using selected SNPs

The selected SNPs representing 10 pseudomolecules each for A subgenome and B subgenome following the above mentioned criteria were subjected to *in silico* validation. The *in silico* validation of the assay involved preliminary screening of the designed array file for each selected SNP, including their p-convert values generated using Affymetrix power tool (APT) AxiomGTv1 algorithm to ensure a high-quality final array (http://www.affymetrix.com/estore/partners_programs/programs/developer/tools/powertools.affx). Both forward and reverse probes of each SNP were assigned with p-convert values, derived from a random forest model to predict the probability of SNP conversion on the array. The model considers factors including the probe sequence, binding energy, and expected degree of non-specific hybridization to multiple genomic regions. SNP probes with high p-convert values are expected to convert on the SNP array with a high probability. Potential probes were designed for each SNP in both the forward and reverse direction, each of which was designated as ‘recommended’, ‘neutral’, or ‘not recommended’ based on p-convert values through which the SNP data sets were easily filtered. A SNP marker/strand is recommended if: p-convert >0.6, no wobbles, and poly count = 0. In other words a SNP marker/strand was not recommended if they had duplicate count >0 or poly count >0 or p-convert <0.4 or wobble distance <21, or wobble count > = 3. Therefore, if a marker has the same recommendation for each strand, then it was tiled on strand with the highest p-convert value. None of the [A/T] or [C/G] markers were selected as they take up twice as many features. Finally, probes for selected SNPs were designed and successfully synthesized on the array chip.

### Genotyping with the SNP array

Affymetrix GeneTitan^®^platform was used to genotype “Reference Set” with the SNP array. Initially the target probes were prepared using each DNA sample having minimum quantity of 20 μL of good quality DNA and 10 ng/μL concentration. This procedure is explained in detail in Affymetrix Axiom^®^ 2.0 Assay Manual. These samples were then amplified, fragmented and hybridized on chip followed by single-base extension through DNA ligation and signal amplification. This procedure is explained in detail in Affymetrix Axiom^®^ 2.0 Assay Manual Target Prep Protocol QRC. The GeneTitan^®^ Multi-Channel Instrument was then used for staining and scanning the samples and the details are provided in (http://media.affymetrix.com/support/downloads/manuals/axiom_2_assay_auto_workflow_user_guide.pdf).

### SNP allele calling and data analysis

Allele calling was done using Axiom™ Analysis Suite version 1.0 using its three workflows i.e., *Best Practices, Sample QC, Genotyping* and *Summary Only* (http://media.affymetrix.com/support/downloads/manuals/axiom_analysis_suite_user_guide.pdf). We used ‘*Best Practices*’ workflow to perform quality control (QC) analysis of samples to select only those samples which passed the QC test for further analysis. The ‘*Sample QC*’ workflow was then used to produce genotype calls for the samples which passed QC analysis using ‘*Best Practices Workflow*’. The ‘*Genotyping*’ workflow was used to perform genotyping on the imported CEL files regardless of the sample QC matrix. Before making the genotyping calls, samples not passing the QC were removed as their inclusion may reduce the quality of the analyzed results. Finally the ‘*Summary Only*’ workflow was used to produce a summary containing details on the intensities for the probe sets for use in copy number analysis tools. It also allows to export the SNP data after the analysis is completed for downstream analysis. We have analyzed the diploid and tetraploid genotypes separately keeping the DQC > 0.75 and call rates >90. The above criteria helped in removing the SNPs having low call rates and keeping only the high quality SNPs for the further analysis. NR database was used to annotate all SNP containing genes using BLASTx program (http://blast.ncbi.nlm.nih.gov/blast/Blast.cgi?PROGRAM=blastx&PAGE_TYPE=BlastSearch&LINK_LOC=blasthome) with cut off E value <1.0E-5. Further, these SNPs were annotated and their effect on gene function was predicted using SNPEff V4.2^34^ software. For this, a file containing reference genome sequence in FASTA format and general feature format (GFF) file containing co-ordinates of various gene features such as, coding sequence (CDS), 5′ untranslated region (5′ UTR), 3′ untranslated region (3′ UTR), etc. were downloaded from the PeanutBase^35^ and used to build genome database. The annotation of gene models identified from peanut genome has been described in Bertioli *et al*.^17^.

### Diversity analysis

Called allelic data was used for studying genetic diversity and genetic relationship among individuals of the “Reference Set”. The polymorphic information content (PIC), major allele frequency, number of observations, availability and gene diversity were calculated using the software PowerMarker ver. 3.25^36^.

### Identification of subspecies specific high frequency SNPs

In order to identify subspecies specific SNPs, the genotypes from the ‘Reference Set’ were divided into three categories viz. *fastigiata, hypogaea* and wild accessions. The allele frequency at each SNP site within each category was calculated. Further, the allele frequencies at each SNP position were compared between three categories and SNPs with contrasting allele frequencies in all the three categories were identified. Additionally, the annotations of these SNPs and their effect on genes were predicted using SNPEff V4.2^34^ and SNPs with moderate (missense mutations) and high (nonsense mutations) effect were identified. Further, the protein coding sequences of genes containing missense and nonsense SNPs were extracted and the functional annotation and gene ontology analysis was carried out using BlastGO^37^ software to determine their involvement in particular biological function.

## Additional Information

**How to cite this article**: Pandey, M. K. *et al*. Development and Evaluation of a High Density Genotyping ‘Axiom_*Arachis*’ Array with 58 K SNPs for Accelerating Genetics and Breeding in Groundnut. *Sci. Rep.* **7**, 40577; doi: 10.1038/srep40577 (2017).

**Publisher's note:** Springer Nature remains neutral with regard to jurisdictional claims in published maps and institutional affiliations.

## Supplementary Material

Supplementary Tables 1-5,7-8

Supplementary Table S6

Supplementary Figure S1

## Figures and Tables

**Table 1:** Genomic position of the selected SNPs for Axiom_*Arachis* array.

SNP categories	A subgenome	B subgenome	Both genomes
Synonymous_coding	12,087	10,137	22,224
Non_synonymous_coding	3,462	3,024	6,486
Intron	11,236	11,986	23,222
Stop_gained	105	80	185
Stop_lost	5	3	8
Intergenic	0	214	214
Start_lost	8	8	16
Non_synonymous_start	3	4	7
Non_synonymous_stop	21	17	38
UTR_3_prime	2,038	1,989	4,027
UTR_5_prime	923	674	1,597
Others	95	114	209
Total	29,983	28,250	58,233

**Table 2:** Genome-wide SNPs selected from A and B subgenomes for development of Axiom_*Arachis* SNP array and polymorphic SNPs identified in ‘Reference Set’.

Pseudomolecules of A and B subgenome	Total SNPs on the array	Average SNPs/1 Mb region	Polymorphic SNPs in tetraploid accessions of the ‘Reference Set’	Polymorphic SNPs in diploid accessions of the ‘Reference Set’	Common polymorphic SNPs between tetraploid and diploid accessions of the ‘Reference Set’	Total polymorphic SNPs in the ‘Reference Set’	Average SNPs/1 Mb region
A01	4,714	41.49	379	3,549	297	3,631	33.62
A02	3,167	33.34	736	2,202	499	2,442	25.71
A03	3,478	25.57	558	2,554	333	2,779	20.43
A04	2,693	21.54	617	1,962	435	2,144	17.15
A05	2,624	23.64	475	1,895	303	2,068	18.63
A06	2,764	24.25	487	1,975	308	2,156	18.91
A07	2,303	28.78	406	1,659	243	1,822	22.77
A08	2,921	57.27	401	2,156	244	2,315	45.39
A09	2,790	22.87	483	2,018	298	2,203	18.06
A10	2,529	22.78	346	1,854	204	1,999	18.00
*Total A subgenome*	29,983	30.15	4,888	21,824	3,164	23,559	23.87
B01	2,405	17.30	361	1,569	181	1,750	12.59
B02	3,112	28.29	617	2,076	400	2,293	20.85
B03	3,443	24.95	523	2,362	287	2,598	18.96
B04	2,588	19.17	516	1,763	311	1,971	14.60
B05	2,576	17.06	395	1,682	204	1,876	12.43
B06	2,793	20.24	433	1,903	236	2,101	15.22
B07	2,638	20.61	384	1,742	193	1,933	15.10
B08	2,671	20.39	323	1,836	180	1,980	15.11
B09	3,152	21.15	479	2,076	270	2,286	15.34
B10	2,872	20.81	393	1,881	199	2,077	15.05
*Total B subgenome*	28,250	21.00	4,424	18,890	2,461	20,865	15.53
Total	58,233	25.58	9,312	40,714	5,625	44,424	19.50

**Table 3:** Summary of species wise SNPs distribution and their features in the ‘Reference Set’.

Species	Ploidy (genome)	SNPs on the array	Polymorphic SNPs	Monomorphic SNPs	% polymorphism
*A. hypogaea*	Tetraploid (AB)	44,501	34,183	10,318	76.8
*A. cardenasii*	Diploid (A)	2,195	1,643	552	74.9
*A. duranensis*	Diploid (A)	3,834	2,912	922	76.0
*A. stenosperma*	Diploid (A)	2,389	2,096	293	87.7
*A. magna*	Diploid (B)	2,605	1,367	1,238	52.5
*A. batizocoi*	Diploid (B)	2,709	2,223	486	82.1
Total SNPs		58,233	44,424	13,809	76.3
