# Supplementary material for: Development and Evaluation of a High Density Genotyping ‘Axiom_Arachis’ Array with 58 K SNPs for Accelerating Genetics and Breeding in Groundnut
Source: Sci Rep. 2017 Jan 16;7:40577. doi: 10.1038/srep40577 (PMC5238394; doi:10.1038/srep40577)
Supplement: Supplementary Figure S1 [file srep40577-s3.doc]

**Development and Evaluation of a High Density Genotyping ‘Axiom_*Arachis’* Array with 58K SNPs for Accelerating Genetics and Breeding in Groundnut**

Manish K. Pandey^1^, Gaurav Agarwal^1,2^, Sandip M. Kale^1^, Josh Clevenger^2^, Spurthi N. Nayak^1^, Manda Sriswathi^1^, Annapurna Chitikineni^1^, Carolina Chavarro^3^, Xiaoping Chen^4^, Hari D. Upadhyaya^1^, Manish K. Vishwakarma^1^, Soraya Leal-Bertioli^3^, Xuanqiang Liang^4^, David J. Bertioli^3^, Baozhu Guo^5^, Scott A. Jackson^3^, Peggy Ozias-Akins^2^, Rajeev K. Varshney^1,6,*^

^1^International Crops Research Institute for the Semi-Arid Tropics (ICRISAT), Hyderabad, India

^2^University of Georgia (UGA), Tifton, GA, USA

^3^Center for Applied Genetic Technologies, University of Georgia (UGA), Athens, GA, USA

^4^Crops Research Institute (CRI), Guangdong Academy of Agricultural Sciences (GAAS), Guangzhou, China

^5^Crop Protection and Management Research Unit, USDA-ARS, Tifton, USA

^6^The University of Western Australia, Crawley, Australia

*Corresponding author: [r.k.varshney@cgiar.org](mailto:r.k.varshney@cgiar.org);

Rajeev K Varshney

International Crops Research Institute for the Semi-Arid Tropics (ICRISAT)

Hyderabad - 502 324, India

Telephone: 91-40-30713305;

Fax: 91-40-30713074


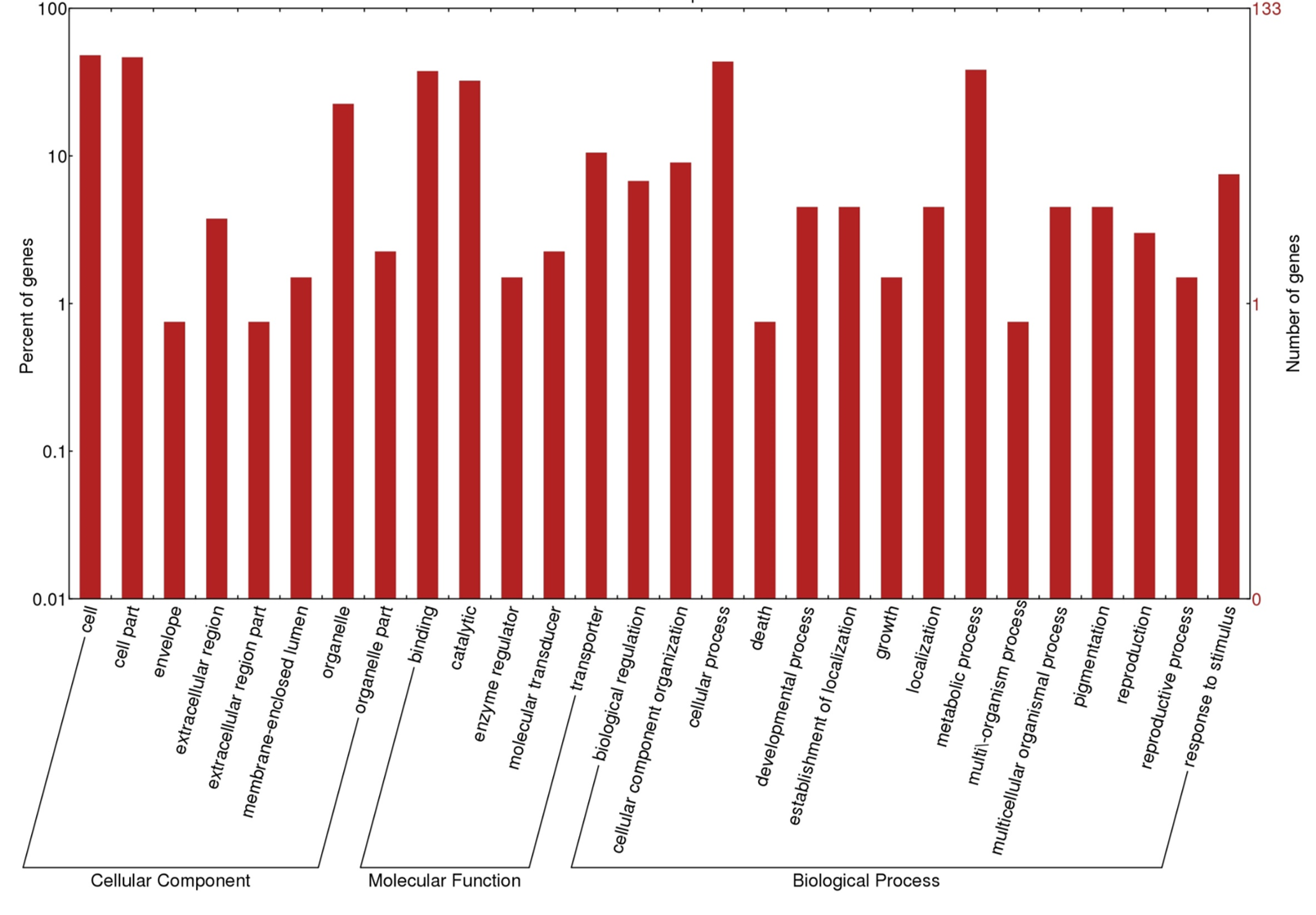


**Supplementary Figure S1: Enrichment analysis of 133 genes with miss-sense or non-sense mutations.** The figure shows percentage of genes identified for three categories of GO terms namely Cellular Component, Molecular Function and Biological Process.
